# Cerebellar infarction caused by vertebral artery dissection: A case report

**DOI:** 10.1097/MD.0000000000034033

**Published:** 2023-06-16

**Authors:** Jing Zhao, Bin Luo, Xinlu Yao, Xiaoyun Zhang, Daquan He, Lina Cai, Yahui Xu, Qin Li, Zhirong Wan

**Affiliations:** a Department of Neurology, Aerospace Center Hospital, Beijing 100049, China.

**Keywords:** case report, cerebellar infarction, high-resolution magnetic resonance imaging, posterior circulation ischemia, vertebral artery dissection

## Abstract

Vertebral artery dissection is an important cause of posterior circulation ischemic stroke in young and middle-aged people. We reported a young man with cerebellar infarction caused by dissection of the right vertebral artery.

**Patient concerns:**

A 34-year-old man presented with intermittent dizziness, blurred vision, nausea, and transient tinnitus 10 days before admission. All these symptoms were gradually aggravated and followed by vomiting and unfavorable movement of the right limbs. All these symptoms gradually aggravated.

**Diagnosis:**

Neurological examination on admission showed ataxia of the right limbs. Magnetic resonance imaging of the head revealed a right cerebellar infarction. High-resolution vessel wall magnetic resonance imaging showed dissection of the right vertebral artery. Whole-brain CT digital subtraction angiography revealed occlusion of the third segment (V3) of the right vertebral artery. This supports the diagnosis of vertebral artery dissection.

**Interventions:**

The patient received anticoagulant treatment with warfarin.

**Outcomes:**

After 2 weeks of treatment, the patient showed remarkably alleviated dizziness and unfavorable movement of the right limbs. After 3 months of treatment, the modified Rankin Scale score was 0. MRI of the head revealed that the original right cerebellar focus was softened, and there were no newly formed infarct foci.

**Lessons:**

When young and middle-aged patients without atherosclerotic risk factors encounter sudden dizziness, tinnitus, and unfavorable limb movement, vertebral artery dissection may be considered. Careful inquiry into the medical history may help make a final diagnosis. Further high-resolution vessel wall magnetic resonance imaging is an effective means to find arterial dissection. Early diagnosis and treatment for vertebral artery dissection has a favorable prognosis.

## 1. Introduction

Vertebral artery dissection (VAD) accounts for 2% of ischemic strokes. However, 10% to 25% strokes in the young are caused by VAD.^[1]^ VAD can be divided into 2 types: spontaneous and traumatic.^[2]^ VAD has diverse clinical manifestations. According to the location of the dissection, the patient may have no symptoms or very atypical symptoms, such as only mild dizziness or headache. The patient may also have posterior circulation ischemia and infarction. In severe cases, clinical symptoms are life-threatening and bring certain difficulties to clinical diagnosis. In recent years, with the development of neurovascular imaging technology, high-resolution vessel wall magnetic resonance imaging (HRMRI) has been used to effectively help diagnose carotid dissection.^[3,4]^ In this paper, we present a retrospective analysis on the clinical diagnosis and treatment as well as imaging data of stroke caused by VAD in a young man, so as to provide reference for clinical work. The patient had provided informed consent for publication of the case

## 2. Case presentation

A 34-year-old man was admitted to our hospital on July 3, 2019 owing to 10 days of intermittent dizziness, which was aggravated and followed by 6 hours of unfavorable movement of the right limbs. On June 23, 2019, the patient was admitted to another hospital because he suffered dizziness of unknown cause, accompanied by blurred vision, nausea, and transient tinnitus of the right ear, which did not affect the daily activity of the patient. No obvious abnormalities were observed on a brain CT scan. The patient was given symptomatic treatment (details unknown). At 6 hours before admission to our hospital, the patient complained of worsened dizziness, nausea, several times of vomiting, clumsy movement of the right limbs and unstable gait, but he denied headache, consciousness disturbance and limb convulsion. In department of emergency in our hospital, diffusion-weighted imaging of the head revealed scattered areas of high signal intensity in the region of the right cerebellum, with corresponding reduced apparent diffusion coefficient values. These findings indicate acute cerebral infarction. The patient was then admitted to department of neurology in our hospital.

### 2.1. Physical examination

The patient’s body temperature was normal. The blood pressure of the right upper limb was 122/ 65 mm Hg, and it was 128/67 mm Hg for the left upper limb. No abnormalities were observed in his heart, lung and abdomen. The patient had a clear mind and could speak fluently. Both pupils were equal in size and round appearance, with d = 3.0 mm. The pupils were sensitive to light. The patient could move both eyes in different directions, without nystagmus. Nasolabial folds were symmetrical. His mouth was not deviated when he showed his teeth. His tongue did not have a tendency to turn away from the midline when extended. Muscle strength was grade 5 in 4 limbs, with a moderate degree of muscle tension, presence of bilateral tendon reflexes, and symmetrical presence of deep and superficial sensations, without pathological indications. The right limbs had a little poor performance in finger to nose test and heel to shin test. The right limbs rotated clumsily. The patient could not stand stably. He was healthy in the past and had no history of smoking or alcohol drinking. He suffered right neck and shoulder pain while barbell squatting 10 days before admission.

### 2.2. Laboratory examination

Laboratory tests were performed in the following aspects: routine blood indicators, blood biochemistry, coagulation function, glycated hemoglobin, homocysteine, and thyroid function (7 items), anti-nuclear antibody, anti-extractable nuclear antigen antibody, antineutrophil cytoplasmic antibody, infection screening (8 items) and tumor markers. All test results were normal.

### 2.3. Imaging examination

**Figure 1.**
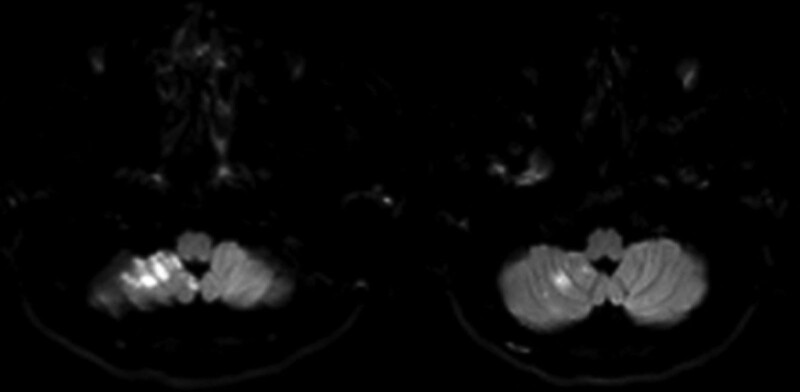
Diffusion weighted imaging scans of the head display scattered patchy areas of high signal intensity in the region of the right cerebellum.

**Figure 2.**
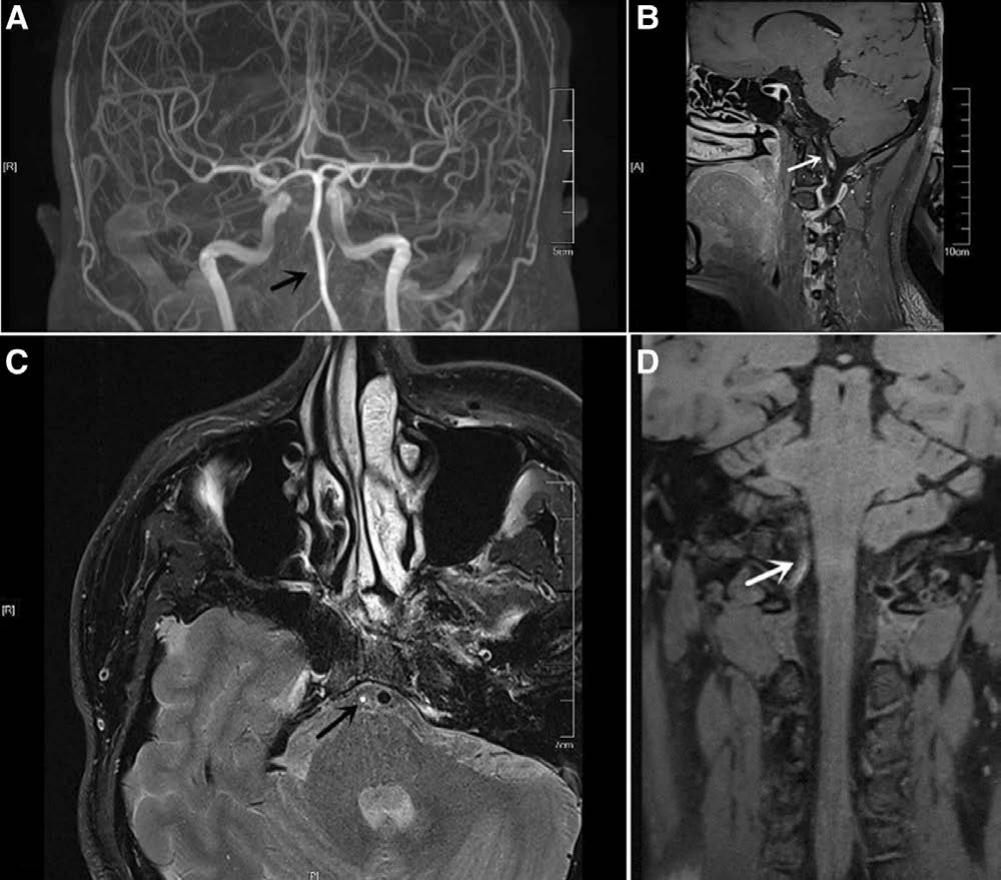
Magnetic resonance angiography scans of the head display occlusion of the right vertebral artery (A). High-resolution vessel wall magnetic resonance imaging reveals intramural hematoma (B) in the right vertebral artery. The hematoma exhibited high signal intensity on T1-weighted images (C). Arterial wall thickening with severe luminal narrowing was observed (D).

**Figure 3.**
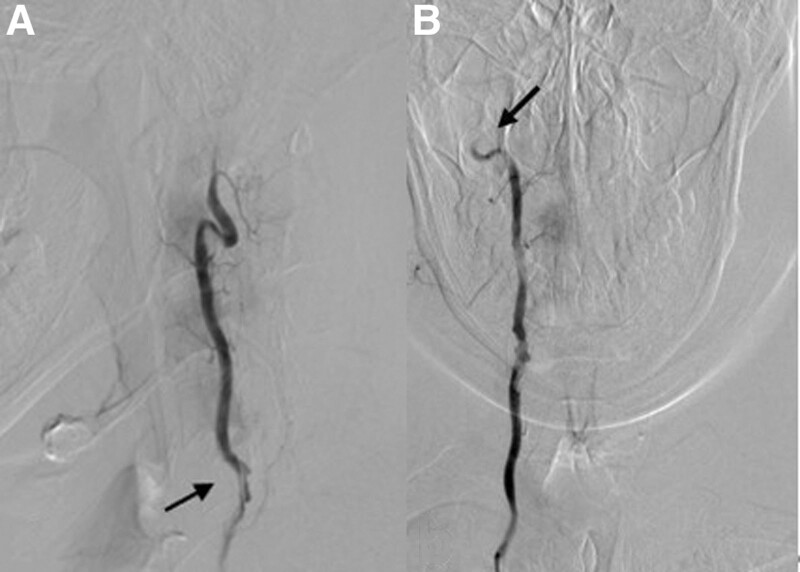
Digital subtraction angiography images reveal irregular morphology of V1 segment (A) of the right vertebral artery and occlusion change of distal V3 segment (B).

Diffusion-weighted imaging of the head showed scattered patchy areas of high signal intensity in the region of the right cerebellum, with reduced apparent diffusion coefficient values (Fig. 1). No abnormalities were observed in the electrocardiogram examination, transesophageal color ultrasound examination, transcranial Doppler color ultrasound and transcranial Doppler bubble ultrasonography. Magnetic resonance angiography revealed occlusion of the right vertebral artery. HRMRI revealed hematoma in the vascular wall of the right vertebral artery, with diffuse vascular wall enhancement and severe luminal stenosis, suggesting arterial dissection (Fig. 2). On DSA images, irregular morphology of V1 segment of the right vertebral artery and occlusion change of distal V3 segment were observed. Arterial dissection was considered (Fig. 3).

### 2.4. Diagnosis

Right cerebellar infarction and dissection of the right vertebral artery were diagnosed.

### 2.5. Treatment

After admission, the patient was given aspirin 100 mg/d and Plavix 75 mg/d. Anticoagulant treatment with warfarin was given after dissection of the right vertebral artery was diagnosed. An international normalized ratio ranging from 2 to 3 was maintained for effective anticoagulation. After 2 weeks of treatment, the patient was discharged with remarkably alleviated dizziness and unfavorable movement of the right limbs.

### 2.6. Follow up

After discharge, anticoagulant treatment with warfarin was continued for 3 months. The modified Rankin Scale score was 0. MRI of the head revealed that the original right cerebellar focus was softened, and there were no newly formed infarct foci. The patient refused angiography.

## 3. Discussion

We reported 1 young man who presented the manifestations of posterior circulation ischemia, including sudden dizziness and tinnitus, which were aggravated with vomiting, unstable standing and clumsy limb movement. The patient was healthy in the past. Physical examination showed that he manifested ataxia of the right limbs, which corresponded to the right cerebellum. MRI of the head suggested ischemic lesion of the right cerebellum. Therefore, cerebral infarction was diagnosed. Then we investigated the underlying pathological mechanism. According to the Trial of Org 10172 in Acute Stroke Treatment Classification, ischemic strokes are divided into 5 subtypes: large-artery atherosclerosis, cardioembolism, small-vessel occlusion, stroke of other determined etiology, and stroke of undetermined etiology. For this patient, large-artery atherosclerosis, cardioembolism, small-vessel occlusion were basically excluded. Considering the pathological mechanism of stroke in the young, because the patient has no genetic family history of VAD and no manifestations of systemic diseases, so hereditary metabolic disease and hereditary small vessel disease are not considered temporarily. The patient’s autoimmune disease antibody and inflammatory indicators are normal, and thus there is no strong evidence to support the diagnosis of immune disease and infectious disease. The patient has no history of drug abuse and tumor development, so toxic and tumor diseases are also excluded. Therefore, we address on non-atherosclerotic vascular diseases, including arterial dissection, fibromuscular dysplasia, moyamoya disease, and cerebral vasoconstriction syndrome. An inquiry into the patient’s medical history showed that the patient suffered right neck and shoulder pain while barbell squatting 10 days before admission. Thus, dissection of the right vertebral artery caused by sports is considered. Subsequently, VAD was confirmed by HRMRI and DSA. Thus, cerebellar infarction caused by VAD was diagnosed. The pathogenesis of cerebellar infarction caused by VAD is that the arterial dissection gradually tears upward from the distal end, and arterial lumen gradually became narrow until occlusion, which was followed by clinical manifestations of ischemic infarction. The morphological changes in vascular wall from V1 segment to V3 segment can explain the aggravation progress of clinical symptoms in this patient.

The pathogenesis of VAD is that the rupture of the intima of the arterial wall causes blood to enter the vascular wall, which makes the vascular wall layered.^[5]^ VAD can be divided into subintimal dissection and subadventitial dissection according to the location of dissection. The former causes lumen stenosis, thromboembolism and posterior circulation infarction. The latter often appears in the form of aneurysmal expansion, leading to local compression or subarachnoid hemorrhage. The etiology of VAD can be divided into 2 types: traumatic and spontaneous.^[6]^ The latter is more common. Slight movements, such as neck hyperextension, hyperflexion and even head turning, may cause VAD. This patient had right neck and shoulder pain while barbell squatting before onset of VAD. Therefore, it is likely that VAD was caused by neck compression.

A review report on arterial dissection shows that dissection of the vertebral artery and intracranial artery are more common in Asian populations, while dissection of the carotid artery and extracranial artery are more common in Caucasians.^[7]^ In this patient, V1 to V3 segments of the vertebral artery were involved, and involvement of such a large range of vertebral artery is related to intracranial progression after V1 segment damage. The clinical manifestations are different due to different affected sites. Headache and neck pain are one of the most common clinical manifestations of VAD, which occur in 65% to 73% of patients. This occurs possibly because of the direct tearing of the vascular wall or the inflammatory stimulation to perivascular nerves.^[8,9]^ As the disease progresses, VAD reduces the blood supply to the cerebellum and brainstem, leading to the occurrence of ischemic infarction.^[10]^ Although most of the ischemic infarcts caused by dissection result from early thromboembolism, some ischemic infarcts form owing to the influence of hemodynamics.^[11]^ Patients may have vertigo, ataxia, fine motor coordination disorder, facial and trunk sensory abnormalities, motor impairment, or Horner’s syndrome. This patient suffered neck and shoulder pain before onset of VAD, and then presented with dizziness, tinnitus, nausea, vomiting, and ataxia of the right limbs. Intramural hematoma in VAD causes lumen stenosis, resulting in hemodynamic disorder. In addition, local emboli fell off, leading to cerebellar infarction.

HRMRI can directly display the lumen of arterial dissection, provides the imaging characteristics of vascular wall lesion, is noninvasive, can be repeated, and provides high-resolution images, all of which effectively improve the diagnosis accuracy for VAD.^[12]^ HRMRI of VAD showed crescent shaped intramural hematoma on T1WI and intimal valve on T2WI.^[13,14]^ Vascular wall enhancement, intraluminal hyperintensity and double lumen sign were observed after enhanced scanning. DSA remains the gold standard for the diagnosis of VAD. Irregular stenosis or occlusion of the lumen, thin line sign, rat tail sign and pseudoaneurysm are the diagnostic signs of VAD. In this case report, HRMRI showed intramural hematoma in V1 segment, diffuse thickening of the wall in V3 and V4 segments, diffuse enhancement of the intima of the wall on enhanced scans, and severe stenosis of the lumen. All these imaging findings are consistent with the findings reported previously.^[4,15]^ In this case report, DSA images showed irregular stenosis of V1 segment of the right vertebral artery, and occlusion of the V3 segment and distal part, all of which further support the diagnosis of VAD.

Recently, a randomized study on antiplatelet treatment *versus* anticoagulant treatment for preventing stroke and death in patients with symptomatic extracranial carotid and vertebral artery dissection, and results showed no significant difference between the 2 treatments.^[16]^ When recurrent ischemic symptoms, obvious vascular stenosis or floating thrombus occur, anticoagulant therapy was recommenced to be preferred.^[17]^ The risk of subarachnoid hemorrhage has been reported to be increased after anticoagulant treatment for intracranial segment VAD.^[18]^ In this report, the patient suffered symptomatic extracranial segment lesion, with obvious artery stenosis. Therefore, anticoagulant treatment with warfarin was given for 3 months. The prognosis was good. The original lesions on cranial MRI were found to be softened. Unfortunately, the patient did not undergo angiography, and recanalization of the artery dissection was unknown.

## 4. Conclusion

VAD is one of the main causes of stroke in young and middle-aged people. Its clinical manifestations are complex and diverse and lack specificity. Timely imaging examination can reduce or avoid missed diagnosis. HRMRI is safe and noninvasive. It is an ideal technique to evaluate vascular wall structure and plaque characteristics. Drug treatment, including anticoagulant and antiplatelet treatments, is the preferred treatment for VAD.

## Author contributions

**Conceptualization**: Zhirong Wan.

**Data curation:** Jing Zhao, Bin Luo, Xiaoyun Zhang, Lina Cai.

**Formal analysis:** Jing Zhao, Bin Luo, Xinlu Yao, Xiaoyun Zhang, Daquan He, Lina Cai, Qin Li.

**Investigation:** Jing Zhao, Daquan He, Yahui Xu, Zhirong Wan.

**Methodology:** Xinlu Yao, Xiaoyun Zhang, Lina Cai, Yahui Xu, Qin Li.

**Resources:** Xiaoyun Zhang, Daquan He, Qin Li.

**Software:** Xinlu Yao, Daquan He, Yahui Xu, Qin Li.

**Supervision:** Bin Luo, Xinlu Yao, Lina Cai, Yahui Xu.

**Visualization:** Zhirong Wan.

**Writing – original draft:** Jing Zhao, Bin Luo.

**Writing – review & editing:** Zhirong Wan.
